# Blood proteomics for quantitative biomarkers of cellular therapies

**DOI:** 10.1186/s40364-025-00837-4

**Published:** 2025-09-29

**Authors:** Philip R. Gafken, Sophie Paczesny

**Affiliations:** 1 Proteomics & Metabolomics Shared Resource, Fred Hutchinson Cancer Center, Seattle, WA United States of America; 2 Department of Microbiology and Immunology, Medical University of South Carolina, Charleston, SC United States of America

## Abstract

Cellular therapies for several blood cancers particularly of lymphoid origin have made remarkable leaps forward. In parallel, blood proteomics, specifically quantitative proteomics, has been a powerful tool for identifying and quantifying protein biomarkers associated with cellular therapies, providing insights into treatment efficacy and toxicity. Both mass spectrometry (MS)-based proteomics and large-scale affinity-based platforms such as Olink and SomaScan have been increasingly implemented in research and clinical laboratories to identify and quantify candidate biomarkers in the blood. Biomarkers are used for risk stratification, early diagnosis, prognosis, and for treatment response prediction and monitoring in context of treatment efficacy and toxicity. These biomarkers might facilitate timely and selective therapeutic intervention and understand pathogenesis mechanisms of responses and adverse events. They are anticipated to undergo faster transition from bench to bedside soon. This review article summarizes recent technical progresses in clinical proteomics. The review also provides current information on validated biomarkers in the field of cellular therapies.

## Introduction

Proteins are biomolecules that better bridge the gap between genomic information and biologic functions and disease phenotypes. Proteins work as part of major biological processes mediating protein interactions that control signaling pathways, cellular processes, and metabolic systems in health and diseases. Proteins represent intermediate phenotypes for disease and provide insight into how genetic and non-genetic risk factors are mechanistically linked to clinical outcomes. Indeed, many diseases, including cancer, are regulated at the protein level emphasizing the importance of the field of proteomics.

Proteomics refers to the large-scale identification and quantification of proteins in biological specimens. Accurate protein identification and quantification are essential for elucidating biological processes underlying health and disease. Contemporary proteome research places strong emphasis on biomarker discovery and validation, aiming to improve early disease diagnosis, stratify susceptibility, predict prognosis, and guide therapeutic decisions. Advances in sample preparation, quantitation strategies, mass spectrometry (MS) instrumentation, and data analysis have been instrumental in addressing clinical questions and accelerating the identification of clinically relevant biomarkers. Over the past few decades, MS-based proteomics has enabled the discovery of thousands of candidate biomarkers across a wide range of diseases. The U.S. Food and Drug Administration (FDA) has approved several MS-based in vitro diagnostic assays, including those for pathogen identification, newborn screening, therapeutic drug monitoring, and vitamin D quantification [1]. Although no FDA-approved MS-based assays currently exist for cellular therapy, several candidate biomarkers identified through MS approaches have been validated using CLIA-certified immunoassays, and some are undergoing evaluation in clinical trials.

Affinity proteomics has emerged as an attractive alternative to MS-based identification of proteins by conducting classical immunoassays at higher throughput and higher sensitivity and in multiplex format. These multianalyte assays rely on advances made in DNA technologies and measure multiple proteins simultaneously in one sample by miniaturizing the assays. The technology implemented by Olink (Uppsala, Sweden) uses proximity extension assays. Detection of a given protein requires binding of two separate antibodies that carry complementary oligonucleotide tags: when two antibodies bind to the target protein, these oligonucleotides can be hybridized and extended by a DNA polymerase. This assay utilizes protein-specific binding properties to generate a readout that relies on DNA concentrations that can be quantify by quantitative PCR [2]. SomaLogic (Boulder, CO, USA) uses an in-house developed library of modified aptamers for highly multiplexed protein profiling of up to 11,000 aptamers. The proprietary assay processes 90 samples per batch, and its readout is on DNA arrays. Instead of matching two binding reagents for increased specificity, the selectivity of the platform is built around specific aptamers selected for their capability to bind to their target protein for an extended time (slow off rate) [3, 4].

This review will first discuss how progresses in MS related technologies, sample preparation methods, labeling reagents, stable isotope labeling reagents, and bioinformatics have led to identification and quantification of several thousand proteins in one experiment with steadily improved sensitivity, resolution and specificity, propelling proteomics into the clinic. Furthermore, large affinity-based methods for plasma biomarkers discovery relying on next-generation proteomics platforms such as Olink and Somascan have been developed in parallel [5], and presently used in clinical trials, will be discussed in the review as well as comparative discussion of the available methods. Integrative systems biology tools will be reviewed as well. Finally, implementation of proteomics into the clinic and metrics used in this setting will be discussed.

## Current methods and technologies

### Mass spectrometry (MS)-based methods

The predominant technology for protein biomarker discovery typically involves mass spectrometry (MS)-based approaches. While other technologies such as microarray, aptamer, and proximity extension assay technologies are useful for protein biomarker discovery, MS is often favored for multiple reasons. First, MS detects proteins in an unbiased manner with essentially no restrictions on what kinds of proteins that can be detected, and it can detect proteins that are not initially targeted or expected at the beginning of an experiment. Second, MS is very sensitive with it being able to detect proteins at low concentrations, which is critical for potential biomarkers that might be present in trace amounts. Third, the technology can provide quantitative results making it possible to compare protein levels between healthy and diseased states or different conditions. The mass spectrometer hardware itself along with sample preparation and data analysis software are under continuous advancement which further bolsters the technologies application in biomarker discovery. Therefore, the technique is preferred in biomarker discovery for its combined advantages in multiplexing capacity and remarkable analytical specificity and sensitivity. Here, we cover current MS methods and highlight recent developments in the field that are improving the depth and quality of identified biomarker candidates.

#### Sample preparation

Blood plasma is an ideal source for potential biomarkers, being the most collected biofluid globally. Blood plasma is relatively easy to collect with blood draws being less invasive than obtaining biopsies from tissues, making it a practical choice for large-scale testing and routine studies. Collection and handling protocols for plasma are well established which maintains consistency and reliability in biomarker discovery experiments. With blood plasma circulating through the bloodstream, it can reflect the overall physiological state of the body, integrating signals from different tissues and organs. While these attributes make blood plasma a powerful source for identifying new diagnostic, prognostic, and therapeutic biomarkers, MS has historically struggled with obtaining deep proteomic analysis of plasma.

Discovery MS-based proteomic analysis of neat plasma is inherently challenging because the most abundant 22 proteins in plasma account for 99% of plasma’s protein content and the range of protein concentrations spans over 10 orders of magnitude [6]. This, in turn, results in only the approximately 300 most abundant proteins being reliably quantified. To increase the number of plasma proteins identified, high abundant protein depletion strategies have been developed which allows for the reproducible quantification of over 1,000 proteins. Plasma depletion for proteomics analysis has typically relied on antibody-based affinity removal of the high abundance proteins. Here, antibodies against the two to fourteen most abundant proteins are immobilized on beads in a liquid chromatography column or they are in a spin-column format, and neat plasma is flowed over the beads to capture the high-abundant proteins, with depleted plasma flowing through for collection and subsequent MS analysis. While high-abundant protein depletion has been successfully used for almost two decades in numerous biomarker discovery studies, the depletion strategies can be costly, potentially preventing its use with MS-based proteomics analysis of larger studies. Below, we discuss recent advances that have been made in plasma depletion that have increased the depth of plasma proteomics results, and in one case, has dramatically reduce the costs associated with depletion.

##### Magnetic beads

Various bead-based approaches have been developed to assist in purifying proteins from various matrices to make them compatible with downstream MS analysis. In the popular SP3 (single pot, solid phase sample preparation) method [7], 1 μm-sized magnetic particles with carboxylated polymer surface are mixed with protein mixtures followed by the addition of an organic liquid, such as ethanol or acetonitrile, to a high concentration which results in proteins being immobilized on the bead’s hydrophilic surface. With magnetic manipulation, the captured proteins can be washed to remove contaminating reagents and the proteins can be further prepared “on-bead” using MS compatible methods. Along similar lines, magnetic nanoparticles in the size range of 200–300 nm were recently developed for use in a similar fashion as SP3, but the nanoparticles were designed to capture proteins from plasma. A total of 43 nanoparticles were designed with varying physiochemical properties, enabling capture of distinct protein patterns by each nanoparticle. The original paper describing this technology selected a panel of five nanoparticles that were used on 141 plasma samples from a non-small cell lung cancer study [8]. The workflow, automated for a 96-well format, enabled the detection of over 2,000 proteins across the dataset, with an average of 1664 protein groups identified per sample. This technology has been commercialized by the company Seer into the Proteograph product, which consists of automated sample preparation instrumentation and analysis software to simplify and streamline mass spectrometry-based blood plasma analysis. Subsequent use of the Proteograph product with further optimization and state of the art mass spectrometry instrumentation has demonstrated in plasma the identification of more than 3100 protein groups per sample with just over 4000 protein identifications across all samples [9] and in a technical evaluation of the Proteograph product approximately 4500 proteins groups were identified [10]. An even more striking result from the analysis of secreted proteins from human monocytes into supplemented culture medium (fetal bovine serum), a situation that faces the same dynamic range issue as serum alone, identified just over 10,000 proteins from 14 samples, with just over 5,000 of the identifications from secreted human proteins [11]. The use of nanoparticles has significantly expanded the depth of proteomic coverage, reaching levels previously unattainable in plasma biomarker discovery. However, the widespread adoption of this technology is currently limited by the high capital investment required for the Proteograph hardware and software, as well as the substantial reagent costs. Additionally, the typical requirement for plasma volumes exceeding 200 µL per sample may restrict its use in small animal models or studies with limited sample availability. Future developments aimed at reducing sample volume requirements and assay costs could enhance the accessibility of this powerful platform for the broader biomarker research community.

Similar to the Seer nanoparticle approach, which enriches low-abundance proteins onto magnetic beads while depleting high-abundance proteins prior to mass spectrometric analysis, the company PreOmics has developed a magnetic bead-based solution called ENRICHplus. This kit is available in formats for 12 or 96 samples and includes reagents for both protein enrichment from plasma and downstream sample preparation steps, including protein reduction and alkylation, enzymatic digestion, and peptide purification. According to PreOmics’ marketing materials, the ENRICHplus workflow enables the identification of over 5,500 protein groups across five plasma samples and using 50 µL of plasma per sample. The protocol is compatible with automation using commercially available liquid handling platforms; however, the cost of the kit translates to approximately $150 per sample for enrichment and preparation.

A third bead-based technology called Mag-Net was recently developed for preparing blood plasma samples for mass spectrometry analysis. This method utilizes strong-anion exchange beads for capturing extracellular vesicles (EVs) from plasma, which are membrane-bound particles that include exosomes, microvesicles, and apoptotic bodies, while at the same time minimizing the enrichment of high abundant plasma proteins. Since EVs are actively shed by cells into the bloodstream, they carry molecular cargo that can reflect the physiological or pathological state of their cells of origin. As such, EVs represent a potentially rich source of cell-specific proteins for biomarker discovery and disease profiling. The Mag-Net approach is automatable (the Opentrons OT-2 and ThermoFisher KingFisher liquid handling robots have been used) and initial reports identified over 4000 protein groups from less than 100 µL of plasma [12]. When coupled with a recently released high-performance mass analyzer, this number increased to over 5000 protein groups [13]. From a practical standpoint, the beads used in the Mag-Net protocol are commercially available (ReSyn Biosciences), and although the exact pricing isn’t listed publicly, the per sample cost of the beads is estimated to be under $1.

##### Acid precipitation

A much simpler approach that has emerged for depleting high abundance proteins in plasma is the use of strong acids for protein precipitation. Perchloric acid has a limited history of being used to precipitate proteins and peptides, but it was recently applied to depleting proteins in plasma. The approach involved adding perchloric acid to samples to a final concentration of 3.5% and centrifuging samples and collecting the pellet, resulting in a pellet that is significantly (but not fully) depleted of high abundance plasma proteins. This depletion was tested on 3199 plasma samples from 1141 patients who were hospitalized with COVID-19 and had longitudinal plasma samples collected during their hospitalization. A total of 44 plates containing the samples were analyzed resulting in a total of 3198 proteins being identified and averaging 1343 proteins per run. Using reference samples on each plate, the authors noted that there was little to no batch effect across the 44 plates and the cost of the depletion reagent per sample was 2 to 3 cents, making this approach incredibly cost-effective for large-scale biomarker studies using plasma [14].

#### Global proteomics

Discovery-based global proteomics studies are carried out through a “bottom-up” scheme. Here a sample is subjected to protein isolation followed by digestion with a protease, typically trypsin, into peptides that are analyzed by an analytical configuration that is composed of a liquid chromatography instrument that separates and elutes the peptides directly into a mass spectrometer for detection: this configuration is referred to as LC-MS. The mass spectrometer detects the mass to charge ratio, or m/z, of eluting peptide ion species in an initial round of mass analysis called an MS scan, and the instrument then fragments peptide ion species detected in the MS scan and records the m/z of those fragments in what is called an MS/MS scan. The effort of the mass spectrometer is focused on detecting as many peptide ion species as possible, fragmenting them, and detecting their fragments during the chromatographic elution of the peptides, which is typically on the order of 15 s to 30 s in duration for a single peptide. With mass spectrometer scan rates of about 40 to 200 Hz and analysis times lasting up to two hours in duration, very complex peptide mixtures produce many thousand or even millions of spectra in a single experiment. The resulting spectra are processed through a protein database search algorithm to identify the peptides back to the proteins from which they originated. Additionally, quantitative data are obtained through the data processing resulting in quantitative values that are relative in nature. That is, individual proteins can be quantitatively compared to themselves across samples in a study.

##### Mass spectrometers

Although numerous mass spectrometers are commercially available with various types and combinations of mass analyzers, discovery-based biomarker studies have been primarily carried out with orbitrap-based instruments. Hybrid orbitrap instruments consist of an orbitrap mass analyzer coupled with either a quadrupole mass analyzer or an ion trap mass analyzer to create an instrument with two functional mass analyzers. Tribrid orbitrap instruments couple the orbitrap mass analyzer with ion trap and quadrupole mass analyzers to create an instrument with a total of 3 functional mass analyzers. The configuration of these instruments allows for highly coordinated use of the coupled mass analyzers to provide sensitive, high-resolution MS analysis in the orbitrap of peptide ions and high-speed MS/MS analysis of peptide fragment ions in either the orbitrap or ion trap mass analyzers.

The orbitrap product line has been expanded by combining the orbitrap mass analyzer with a novel time-of-flight (TOF) mass analyzer called Astral (Asymmetric Track Lossless). The Astral analyzer acts as an electrostatic trapping device that uses temporal and spatial focusing of ions across asymmetric ion mirrors with a time-of-flight separation of around 30 m. The Astral achieves a mass resolution of 80,000 (at m/z 524), a scan speed of 200 Hz, and single ion detection sensitivity. As with previous orbitrap instruments, the Astral model is a tribrid design built with orbitrap, quadrupole, and Astral mass analyzers working in a coordinated fashion to optimize the efficient transmission of ions to carry out MS and MS/MS scans [15]. Recently, the Astral has been upgraded through a combination of hardware and software improvements to create the Astral Zoom. Most notable, ions are accumulated at the front end of the instrument to increase sensitivity while also increasing acquisition rates up to 270 Hz in the Astral analyzer. Additionally, the Astral Zoom analyzer can achieve a resolution of 100,000 at m/z 138 making it capable of analyzing multiplexed samples in the TMTpro 32-plex format (see below) [16].

Besides orbitrap-based instrumentation, ion mobility coupled with time-of-flight (TOF) mass analyzers has seen a dramatic growth in popularity for biomarker discovery. The gas-phase separation of ions by ion mobility is carried out by subjecting ions to an electric field that separates them based on their mobility through a gas, such as helium or nitrogen, with the mobility being influenced by the size, shape, and charge of the ion. Ultimately, ion mobility adds an additional dimension of separation to LC-MS, reducing the complexity of ions packets entering the TOF analyzer and increasing the sensitivity of detection. The most notable ion mobility-enabled mass spectrometer used for biomarker discovery is the trapped ion mobility spectrometry (TIMS) TOF instrument. The TIMS-TOF utilizes two sequential TIMS regions with the first region used to trap ions and the second region used for ion mobility separation. In general operation, trapped ions from the first region are separated by their ion mobility in the second region and detected by the TOF mass analyzer for a MS scan. Using the MS scan as a guide, ions are selected serially from the second TIMS region for fragmentation, with the fragment ions being detected in the TOF mass analyzer as an MS/MS scan [17]. Multiple product lines from Bruker utilize the TIMS TOF technology, with the latest generation timsUltra AIP being designed with an ion processor to maximize ion transmission and enhance sensitivity of the instrument. The timsUltra AIP can achieve scan speeds of 300 Hz and a mass resolution of 60,000.

##### Data acquisition methodologies

**Fig. 1:**
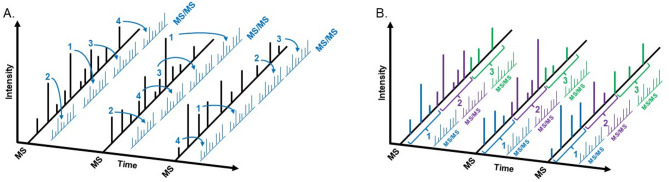
Overview of Mass Spectrometry-based Proteomics Data Acquisition Strategies. **A**. Data dependent acquisition (DDA) selects individual ions from a survey scan, or MS scan, as targets for fragmentation to generate fragmentation scans, or MS/MS scan (note, the z-axis corresponds to m/z, or mass to charge ratio). One MS/MS spectrum is generated from each ion species selected from the MS scan, with the top N precursors being selected before the next MS scan is collected. In this example, four precursors are selected to generate four fragmentation spectra before repeating the process on a new set of ions, but realistically many more ions are typically selected for fragmentation by the mass spectrometer, potentially over 200 ion species. **B.** Data independent acquisition isolates precursor ions over a window of m/z values in the MS scan for subsequent fragmentation. The window is moved across the m/z range of the MS scan followed by subsequent, sequential MS/MS spectra being collected until the desired range of the MS spectrum has been covered. This process is repeated for every MS spectrum that is collected over the course of the chromatographic elution. In this example, three identical ranges (depicted in blue, purple, and green) across each MS spectra are subjected to fragmentation to generate MS/MS spectra. Realistically, many more than 3 windows are used per MS spectrum; with the newest mass spectrometers, over 200 windows may be surveyed per MS spectrum

Data-dependent acquisition (DDA) has traditionally been the primary mass spectrometry (MS) strategy employed in discovery-based biomarker research. This approach dynamically selects precursor ions during the initial MS survey scan, where ions are chosen for fragmentation based on their relative intensity (Fig. 1A). The most abundant ions are prioritized for MS/MS analysis, generating fragmentation spectra used for peptide identification. To minimize redundant data collection, selected ions are temporarily placed on an exclusion list, typically for 15 to 60 s, preventing their repeated selection within that time frame. This cycle continues throughout the duration of the analytical run, enabling broad sampling of the proteome. Overall, this method is called DDA because the selection of ions for further analysis depends on the data collected in real time. While DDA has been widely adopted, it presents two notable limitations that can hinder biomarker discovery. First, the stochastic nature of ion selection leads to reduced run-to-run reproducibility and frequent missing values in quantitative datasets. Second, low-abundance ions are often overlooked, limiting the detection of potentially important biomarkers. To address these challenges, data-independent acquisition (DIA) has emerged as a complementary strategy.

DIA is a discovery-based mass spectrometry method that systematically fragments all ions within predefined mass-to-charge (m/z) windows, typically ranging from 2 to 20 m/z, across the MS spectrum. These windows are sequentially scanned over a specified m/z range during each cycle, and this process is repeated throughout the duration of the analysis (Fig. 1B). Because both the m/z window size and cycle time are consistent across experiments, DIA offers highly reproducible data acquisition. All ions within each window are subjected to fragmentation and MS/MS analysis, enabling comprehensive sampling and the potential identification and quantification of a broader range of peptides, including those of lower abundance. In contrast to data-dependent acquisition (DDA), which preferentially selects the most abundant precursor ions for fragmentation, DIA avoids this selection bias. As a result, it provides a more uniform representation of peptides and significantly reduces the incidence of missing quantitative values. However, the resulting MS/MS spectra are highly complex due to the simultaneous fragmentation of multiple co-eluting peptide species. Advanced computational tools address this complexity by deconvoluting the spectra, matching observed fragment ions to predicted ones, and reconstructing elution profiles to enable accurate quantification. The field of DIA is advancing rapidly with both commercial and open-source software solutions now available for the qualitative and quantitative analysis of DIA data [18], including some employing deep-neural-network strategies [19].

##### Samples per day

Advancements in liquid chromatography instruments have resulted in very stable and reproducible LC-MS analyses. With optimized liquid chromatography systems coupled with mass spectrometers that acquire data at > 100 scans per second, LC-MS run times for obtaining > 8000 proteins quantified in a human sample can occur in less than 10 min. Many MS-based proteomics labs now associate their methods with the number of samples that can be analyzed in a day. For example, they might have methods for 12 samples per day (SPD), 24 SPD, 48 SPD, and higher [14, 20, 21]. Ultimately, this means that sample throughput is dramatically increasing, with faster scanning instruments making large-scale studies easier to consider. In the arena of biomarker discovery, sample cohort sizes can now be expanded to many hundreds or even thousands of samples that can be analyzed by MS in a few months or less (e.g. 2000 samples analyzed by a 48 SPD method would take about 42 days, or 1.4 months, of analysis time while a 180 SPD method would take about 11 days of time). Increased sample numbers in a study will lead to increased proteins identified across the sample set and increased power in the statistical analysis of the results, both welcomed outcomes for having increased throughput in a biomarker discovery project. However, higher throughput often comes at the expense of proteomic depth, potentially overlooking subtle but biologically significant signals. Consequently, mass spectrometry-based biomarker discovery experiments are generally designed to prioritize analytical depth over throughput, focusing on smaller, well-characterized cohorts. In contrast, validation studies shift the emphasis toward throughput, enabling the analysis of much larger sample sets. Ultimately, the optimal balance between throughput and depth depends on factors such as cohort size, acceptable analytical depth, and available resources.

##### Multiplexed quantitative proteomics

While DIA methods are advancing and gaining more utility, these methods are still somewhat specialized for MS labs. The ability to quantitatively analyze multiple samples simultaneously, or multiplex, by MS-based DDA methods has been an active area of proteomic development for over 20 years. Emerging to the forefront of this development has been a chemical labeling approach called Tandem Mass Tags (TMT). In its current form, TMT is a set of isobaric reagents that allows for multiplexing up to 18 samples in a single batch [22], with a recent expansion to multiplexing 35 samples [23]. With the TMT methodology, samples are first proteolytically digested with trypsin to produce peptides, with each sample processed being considered a separate “channel”. Chemical labeling with a unique TMT reagent for each sample is carried out on the digested peptides. The reagent is designed with a NHS-ester moiety at one end of the molecule that covalently binds to primary amines on the N-terminus of the tryptic peptides and to the side chain amine of lysine. After labeling, the samples are combined into one pool and chromatographically fractionated into subpools that are analyzed by LC-MS. Due to the isobaric structures of the TMT reagents, a chemically labeled peptide that is present across all samples is labeled by a different TMT reagent, but the isobaric design of the label means all the labeled peptides have the same mass. After the selection of the combined labeled peptide in MS, MS/MS analysis of the TMT-labeled peptide provides fragment ions that can identify the peptide via a database search algorithm. Additionally, the MS/MS spectrum contains unique reporter ions from each reagent used that fragment from the TMT reagent and appear at approximately m/z 126 through m/z 135. The intensities of these ions are used to determine the relative quantification of the peptide across the various samples being analyzed, with the peptide acting as a quantitative surrogate for the protein it originated from.

Multiplexing with TMT has certain advantages for general proteomics and biomarker discovery experiments. First, multiplexing allows for deep proteomic coverage of up to approximately 8,000 to 12,000 proteins quantified in a single 18plex experiment (e.g. cell lysate, tissue samples), taking approximately 3 days of MS analysis time. From a historical perspective this level of throughput is notable; however, with advanced mass spectrometers that can acquire data at 100 to 300 Hz, this throughput will assuredly increase in the near future. Second, since the samples are prepared simultaneously and mixed together early in the sample preparation scheme, multiplexing minimizes sample handling errors and reduces variability. Third, simultaneous analysis of the multiplexed samples provides higher data quality and more reliable quantification, with fewer missing values amongst the quantification results. Finally, TMT multiplexing does not need to be limited to just 18 samples; if more than 18 samples are part of a study, a common reference sample can be constructed and batches of 17 samples plus one reference sample can be made. The common reference allows for relative quantification across multiple batches, and it also minimizes the variation of the results by allowing for batch effect correction.

TMT has proven to be incredibly versatile, with it being applied to cell lines and bacterial lysates, tissues biopsies, organoids, and clinical fluids such as plasma, serum, urine, and cerebral spinal fluid. The robustness of this methodology for biomarker research and molecular tumor characterization has been demonstrated in its use by the National Cancer Institute’s Clinical Proteomics Tumor Analysis Consortium (NCI-CPTAC) published studies associated with breast [24, 25], brain [26–28], colorectal [29], ovarian [30–32], lung [33, 34], and endometrial [35, 36] cancers. Furthermore, biomarker studies for the early detection of Alzheimer’s Disease have relied on the TMT multiplexing technology [37, 38]. All these studies have used TMT on tens to hundreds of samples with excellent results reflecting the importance of the TMT technology for discovery-based biomarker discovery.

#### Single cell quantitative proteomics

Biomarkers identified within the tumor cells themselves and the associated tumor microenvironment (TME) (e.g. fibroblasts, endothelium, and immune cells) provide an invaluable biologic context for the development and evaluation of immunotherapies. Understanding interactions of these cells at the single cell level will provide invaluable insights in the biology of the TME. The analysis of single cells by various biophysical techniques has grown dramatically in the past decade, with next-generation sequencing technology (NGS) being widely used in a high throughput manner to measure gene expression via mRNA abundance. However, there is a low correlation between mRNA abundance and protein abundance [39], making mRNA abundance a poor predictor of the biology being carried out at the protein level in a cell. As a result, MS-based methods for carrying out proteomic characterization at the single cell level has been advancing rapidly. To make single-cell proteomics a more accessible technology, advances in sample preparation and MS methods have been implemented.

While the first experiments demonstrating the feasibility of single cell proteomics used standard microfuge tubes and pipettors for liquid dispensing and handling, the development of chip-based methods to contain arrays of samples coupled to sub-microliter robotic liquid handling devices are becoming favored. Chips with hydrophobic surfaces are being designed to focus suspended cells in nanoliter droplets with sample preparation taking place through robotic nanoliter dispensing of reagents directly on to the focused droplet [40, 41]. The chip-based approach minimizes sample loss during sample preparation to increase the number of proteins identified and it also allows for 1000s of droplets containing single cells to be dispensed on the chips to dramatically scale up sample preparation. Both single cell isolation and nanoliter dispensing and mixing of liquids on chips has been automated with the CellenONE robotic platform, with throughputs of over 3000 samples prepared per day recently reported [42].

To keep up with sample preparation throughputs, as well as the need to carry out single-cell experiments at levels of over 10,000 cells, MS methods must have compatible throughputs. The recent advances in data independent acquisition methods (as described above) and increased sensitivity and data acquisition speeds of mass spectrometers have reduced LC-MS injection to injection times to as low at 5 min, or 288 samples per day. Even with a 288 samples per day method, approximately 35 days of total MS acquisition time would be required to analyze 10,000 cells. Currently, the primary solution to increasing MS throughput is to utilize chemical-labeling multiplexing technology. Most notable is the tandem mass tag technology which can multiplex 18 samples into a single experiment, with recent advancements in the technology allowing for multiplexing out to 35 samples in a single experiment [23]. It has been recently demonstrated that using the 32plex single-cell multiplexing approach, a throughput of 1018 single cells per day could be analyzed at a depth of 800 to 1200 proteins identified in each human single cell [42].

In terms of sample preparation, it should be noted that fluorescence-activated cell sorting (FACS) is highly suited for isolating single cells and single cell population into 384-well plates and it has been utilized for single cell proteomics [43]. To date, FACS-based single cell experiments have not demonstrated the throughput of the CellenONE with chip-based platform. The FACS-based approach, however, has been used in a striking application of single-cell proteomics to characterize the cellular hierarchies in an acute myeloid leukemia model. Blast, progenitor, and leukemic stem cells were sorted by FACS and analyzed by mass spectrometry to quantify around 1000 proteins from over 2000 cells to identify functional difference in the cell differentiation stages [44]. This research is an exciting example of the future use of single cell proteomics to studying leukemia and other blood disorders and moves the field from proof of concept and optimization to direct biomedical research application. Beyond high throughput biomarkers, technologies have been developed for a better understanding of the TME biology such as SCoPE-MS (single-cell proteomics by mass spectrometry) that uses a carrier proteome, a sample added at a higher concentration than the single-cell proteome. The carrier proteome, which is labeled with isobaric tags, increases the signal of peptides, allowing for better identification and quantification of proteins in single cells [45, 46].

#### Proteoforms

Proteoforms studies have become a central focus in clinical proteomics to understand diseases and personalized medicine. The term “proteoforms” describes the range of different structures of a protein product of a single gene, including variations in amino acid sequence and post-translational modifications. This variety in protein structure contributes to the biological complexity observed in humans and animals. As the concentration of a particular proteoform increases or decreases in pathogenic states, proteoforms have been used in clinic as biomarkers of diseases. Importantly, within-person reproducibility of proteoforms related to inflammation (i.e. CRP, serum amyloid A, calprotectin (S100A8/9) and renal dysfunction (cystatin C) has been confirmed allowing their use as biomarkers [47].

### Affinity-based methods for plasma biomarkers discovery

New technologies based on affinity reagents for capture and detection of specific proteins are receiving increasing attention in plasma proteomics due to their performance characteristics, cost, and usability. Platforms using paired, nucleotide-labeled antibody probes (Olink) and single-strand DNA aptamer reagents with slow off-rate kinetics (SomaScan) can be automated for efficient multiplexing of thousands of proteins at high sample throughput. While these platforms have streamlined workflows as compared to LC-MS/MS–based methods, this comes at the cost of decreased specificity for molecular characterization [48]. Proteomic profiling with these affinity platforms has already been performed in many studies [49–52]. It is critical to understand the relative strengths and weaknesses of these technologies and compare these platforms to one another to offer an opportunity for high-throughput assessment. Earlier comparisons have been limited by sample size and the number of proteins measured at the time of comparison but did suggest differences in platform characteristics and reproducibility [53]. More recently, a larger effort to compare these platforms demonstrated very poor correlation between a large number of reagents targeting the same protein [54]. While some differences were explained by a variety of platform and protein factors, an assessment of comparative accuracy was only recently performed.

#### Olink

The innovation in the technology of the Olink platforms is the Proximity Extension Assay (PEA) which achieve high levels of specificity and detection sensitivity by combining affinity-based proteomics with next-generation sequencing (NGS) readouts. PEA is an immunoassay technology that uses antibodies attached to unique sequences of DNA oligonucleotides (DNA-tags). Each protein in the sample is targeted by two antibodies with complementary DNA sequences. Due to the proximity, when the two antibodies are attached with the target protein, DNA tags are hybridized and extended making a unique DNA barcode for each protein analyzed. This double-stranded DNA is then amplified using Polymerase Chain Reaction (PCR) and quantified using NGS readout on the Illumina^®^ NovaSeq or Illumina^®^ NextSeq platforms. Olink Explore can be useful for biomarker discovery due to the broad range of proteins (up to ~ 6000 proteins). This technology has preset pairs of antibodies covering disease areas including inflammation, neurology, and oncology.

#### Somascan

SomaScan is a high-throughput, aptamer-based proteomics assay designed for the simultaneous measurement of thousands of proteins with a broad range of endogenous concentrations. In its most current version, the SomaScan assay can measure up to 11000 human proteins simultaneously from a single 55-µL sample of serum, plasma, or other fluid types. It uses SOMAmer^®^ Reagents, single-stranded DNA aptamers, modified to bind to specific proteins with high specificity and sensitivity. The targeted proteins cover a comprehensive range of diseases. SomaScan detects proteins over a broad dynamic range using a tiered dilution approach to measure proteins of very high abundance and very low abundance separately, such that a total range of 10 logs can be measured.

Although intra and inter coefficient of variations (CVs) are acceptable for each platform, the correlation of these two platforms for the same targets is poor. The Olink platform does correlate well with ELISA for specific targets but somascan does not, at least in the earlier version, which seem to have improve recently with the new iteration [53]. Other disadvantages are that both Olink and Somascan requires a minimum of 20 targets to be used while panels with AUC of ROC (AUROC) > 0.8 for prognostic markers of graft-versus-host disease (GVHD) usually requires less than 5 proteins [55–57]. Quantification is relative and not absolute since there is no standard curves like for an ELISA. Therefore, these platforms are preferred for discovery rather than for validation of biomarker panels. In any case, these technologies have limitations. Associations of interest should be supported by independent validation of target specificity and should include consideration of possible cross-reactivity and epitope effects. Table 1 summarizes the attributes of these different plasma proteomics platforms. Importantly, as none of the current methods is the optimal or sole solution, validation across multiple technologies will be key, with the most important findings further supported by cellular and other functional studies.

**Table 1:** Attributes of plasma proteomics platforms

Attributes	Tandem Mass spectrometry (MS/MS)	Olink	Somascan	ELISA
Analytes measured	Peptide MS spectra	Pairs of antibodies	Aptamer	Pairs of antibodies
Unspecific binding	NA	Mitigated by use of two antibodies	Possible, needs to be verified on a case-by-case basis	NA if single analyte
Cross-reactivity	NA	Mitigated by use of two antibodies	Possible, needs to be verified on a case-by-case basis	NA if single analyte
Interference	Possible bias owing to over-representation of peptides from abundant proteins	Possible owing to protein interactions	Interference with DNA-binding proteins has been reported	NA
Sample throughput	Low, dependent on protocol; future automation of workflows expected	High owing to adaptation of workflows and multiplexing	High owing to automation of workflow and multiplexing	Extremely high (24 to 48 h turnaround), several CLIA clinical test
Proteome coverage achievable in high throughput	~ 2,000	~ 3,000	~ 11,000	One at a time or low number of analytes in multiplex < 20
Preparation impacting sensitivity	Protein depletion, peptide fractionation and selection	Selected panels, dilution steps and signal amplification	Selected panels, Multiple sample dilution steps	
Quantification	Relative quant with multiplexing reagents or DIA; absolute quant based on peptide standards	Quantification dependent on technique and based on full-length standards	Quantification based on calibrators	Quantification based on standard curve with set protein concentration values
Target identification	Targeted or based on guidelines defining length and peptide numbers per protein	Targeted approach with selected sets of predefined analytes	Targeted approach with a predefined list of analytes	Highly specific tested for cross-reactivity
Data processing after analysis	Time demanding and dependent on algorithm	Minimal but dependent on technology	Standardized process, proprietary	Widely available readers, data in few minutes
Sustainability	MS spectra can be reprocessed using novel or updated databases	Continued availability of identical antibodies may become a limitation	Unlimited reproducibility of synthetic aptamers	Gold standard for CLIA approved clinical test
Coverage of protein post-translational modifications	Possible using dedicated workflows	Possible owing to use of modification-specific reagents	Requires generation of modification-specific binders, under development	Possible if analyte is a PTM and available kit
Coverage of isoforms	Possible with appropriate protein database search strategy	Not addressed and requires generation of isoform-specific binders and assays	Requires isoform-specific binders, some exist	NA
Accessibility	Most common and versatile proteomics technique	Growing use of multiplexed systems over classical ELISAs	Proprietary technique	Gold standard for CLIA approved clinical test
Cost	Low sample cost but high capital cost and requirement of skilled personnel	High	Higher	Lowest cost per sample

Footnotes: MS, mass spectrometry; DIA, data-independent acquisition; ELISA, enzyme-linked immunosorbent assay; NA, not applicable; CLIA Clinical Laboratory Improvement Amendments; PTM, post-translational modifications

## Systems biology approach for proteomics

Understanding organisms at the proteome level will contribute to the development of logic models since proteins are highly modified due to post-translation modifications (PTMs) and therefore noticeably more diverse as compared to the more static genome. Systems biology constitutes a crossover between knowledge-based modeling and omics data-driven approaches. Bioinformatics is a broad multidisciplinary field which is indispensable for systems biology that deals with omics data, mathematical modelling, and network analysis. This is because the dynamic behaviors of biological systems are beyond human intuitive grasp due to the sheer number of components (biomolecules, cells, drugs, and each other) which interact. System-level understanding is only possible through computational models and simulations. However, the current tools for a global proteome analysis via systems biology study can only use information from total quantifiable proteins in any biological system. Furthermore, protein candidates can be further examined using pathway and network analysis that are approaches that find interacting partners or ligands. All the proteins expressed, and associated interactome information, can be used to design a workable model for a biological system and hence can be tested in systematic series of perturbation experiments.

### Proteogenomics studies

Proteomics has been utilized in an integrative approach, combining other omics such as genomics, transcriptomics, and metabolomics to comprehensively understand biological processes. Rather than investigating isolated parts of genes and proteins in an organism like it is done with the traditional biology method, a system approach characterizes the dynamics and function of a working biological system which could then be tested upon series of classical biological experiments. For example, a recent study looking at the Clinical Proteomic Tumor Analysis Consortium (CPTAC) proteogenomics data from 1,043 patients across 10 cancer types enabled the construction of a comprehensive landscape of protein and peptide targets for companion diagnostics, drug repurposing, and therapy development [58]. In the context of hematopoietic cell transplantation (HCT), T cell alloreactivity against minor histocompatibility antigens (mHAgs)-polymorphic peptides resulting from donor-recipient (D-R) disparity at sites of genetic polymorphisms drives the graft-versus-leukemia (GVL) effect and, unfortunately, graft-versus-host disease (GVHD) reaction as well. Using integration of polymorphism detection by whole-exome sequencing of germline DNA from D-R pairs with organ-specific transcriptional- and proteome-level expression, Cieri et al. could identify the future occurrence of acute GVHD, pulmonary chronic GVHD, and defined promising GVL targets [59].

### Integrative proteomics in network analysis

Network analysis is a method of studying the relationships between the nodes in a network and understanding how the network functions. There is a rapid accumulation of proteomics data in recent years but those are highly variable, with results being sensitive to data preparation methods, sample condition, instrument types, and analytical method. To address this challenge in analysis, several approaches have been developed to incorporate biological function and network topological information to the data. There are four categories of tools available: (1) tools with basic functional information and little topological features such as GO category analysis, (2) tools with rich functional information and little topological features such as GSEA, (3) tools with basic functional information and rich topological features such as Cytoscape, and (4) tools with rich functional information and rich topological features such as PathwayExpress. These tools have been reviewed in [60]. PathwayExpress and derivative have been utilized for the development of diagnostic proteomics markers of pulmonary chronic GVHD [61] and prognostic markers of GVL [62].

## Implementation of proteomics for clinical biomarkers

### NIH and FDA types of biomarker definitions

The ‘Biomarkers, EndpointS and other Tools’ (BEST) Resource was established in 2016 by the FDA-NIH joint leadership council to define and outline biomarker roles in biomedical research, clinical practice and pharmaceutical drug development [63]. Five types of biomarkers are defined: diagnostic, risk, prognostic, predictive and response/monitoring. In the case of GVHD, the definitions infer timing post-HCT. The definitions of type of biomarkers are found in Table 2.

**Table 2:** Definitions of types of biomarkers

Type of biomarker	Definition
Diagnostic	An assay used to confirm the presence of the disease
Risk/Susceptibility	An assay that indicates the potential for developing the disease in individuals who do not have clinically apparent disease
Prognostic	An assay used to identify likelihood of a clinical event, disease recurrence or progression in patients who have the disease
Predictive	An assay used to identify individuals who are more likely than similar individuals without the biomarker to experience a favorable or unfavorable effect from exposure to a specific medical product (before treatment is received)
Response/Pharmacodynamic	An assay used to show that a biological response has occurred in an individual who has been exposed to a medical product (after treatment is received)
Safety	An assay used to indicate the likelihood, presence or extent of toxicity as an adverse effect of a medical intervention

### Biomarker development framework

**Fig. 2:**
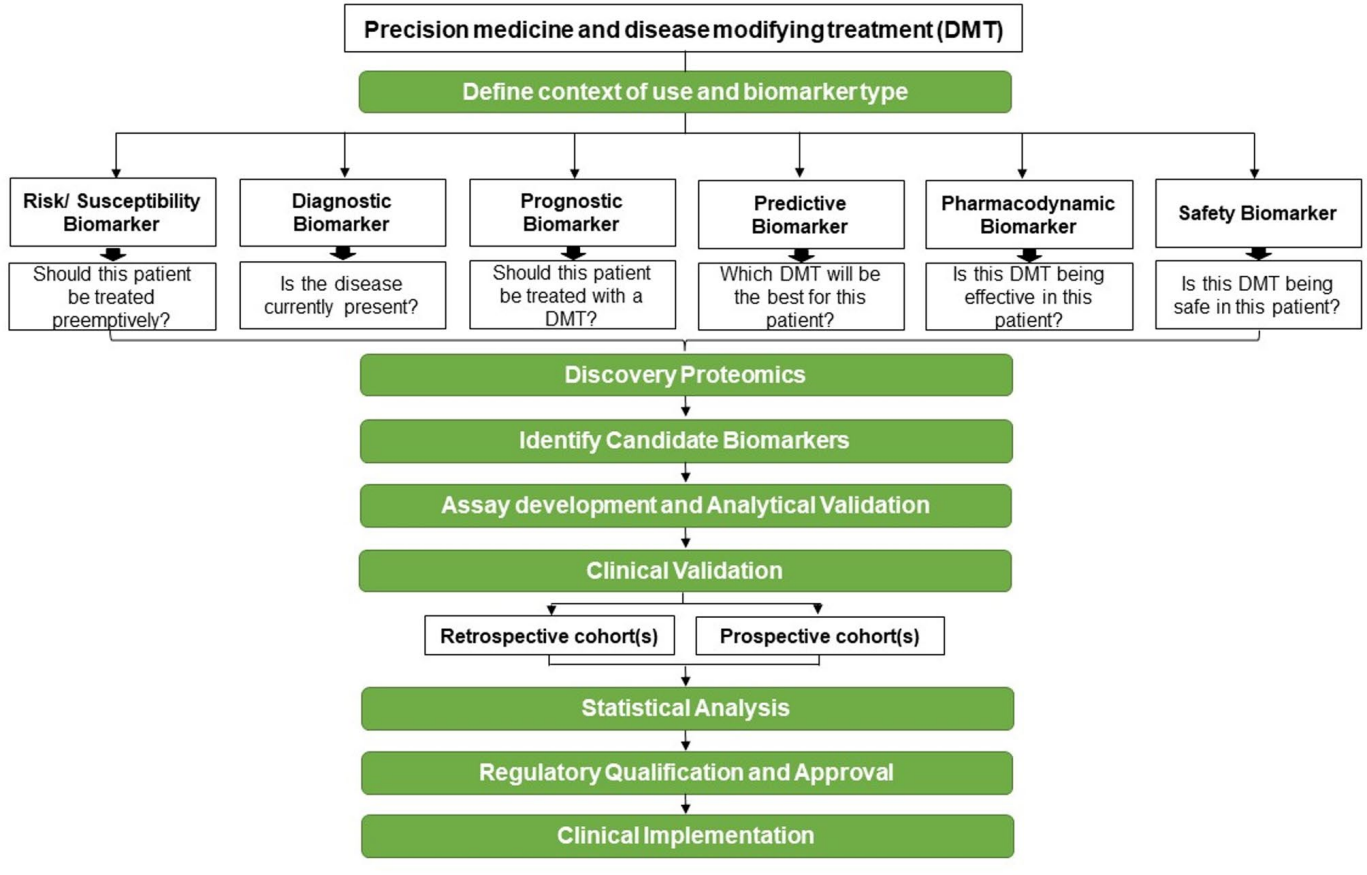
Framework of Biomarker Development. This workflow outlines the key stages in developing biomarkers that pave the way for precision medicine and disease-modifying treatments (DMTs). It begins by clearly defining the *context of use* and specifying the *biomarker type* (see definitions). The process then moves into discovery proteomics, where potential candidates are identified. Next comes assay development and analytical validation, ensuring the biomarker can be reliably measured. This is followed by clinical validation, which involves rigorous testing in at least two independent cohorts—initially retrospective, then prospective. After successful validation and robust statistical analysis in large-scale studies, the biomarker advances to the qualification phase, where it undergoes regulatory review. If approved, the final step is clinical implementation, bringing the biomarker into real-world use for its defined purpose. his workflow represents the recommended steps for biomarker development leading to precision medicine and disease modifying treatment (DMT)

The development of a biomarker is complicated and involves many steps from discovery to implementation in the routine clinical care of patients. Figure 2; Table 3 highlight the important steps of biomarker development. These steps must all be followed to ensure the validity and clinical utility of newly discovered biomarkers. There are several steps involved in the development of a biomarker for clinical use. The first step is a discovery phase that usually compares cases and controls. Candidate biomarkers are proteins selected for their differential expression between cases and controls, a biologic plausibility, and can be run on a high throughput assay such as an ELISA. Once a newly discovered biomarker demonstrates promising statistical validity evaluated with Receiver Operating Characteristic (ROC) curves, validation must be performed on an independent cohort, ideally large and from multiple institutions. Finally, the biomarker should be verified. This is often done in large prospective studies that can also help determine cutoffs for high or low risk for a specific outcome and clinical use.

**Table 3:** Biomarker(s) development phases

Phase	Goal
Discovery	Identify candidate biomarkers through exploratory studies comparing biological samples from cases and controls patients for a specific disease context. Discovery platforms include MS/MS, Olink or Somascan.
Analytical Validation	Assess the reliability and accuracy of the methods used to measure the biomarker ensuring the assay provides consistent and accurate results.
Clinical Validation	Determine the clinical utility and relevance of the biomarker for the specific context and biomarker type. This phase involves large-scale clinical cohorts (retrospective, independent, prospective) to evaluate how well the biomarker performs in a real-world clinical setting.
Clinical Application	Integrate the validated biomarker into clinical practice using the biomarker to guide patient management.
Biomarker Qualification	This is a formal regulatory process to recognize the biomarker’s utility for use in specific drug development. This involves demonstrating to the US Food and Drug Administration (FDA) the biomarker’s performance characteristics and establishing its context of use in almost all case associated with a drug.
Clinical implementation	If the biomarker has been qualified by the FDA, Integrate the qualified biomarker with the specific drug into clinical practice (i.e. PD1 staining with a threshold for a checkpoint inhibitor (still pending).

### Validated blood proteomics biomarkers for cellular therapies

Biomarkers that have the greatest support for their validity for risk, diagnosis, and prognosis prediction for toxicity and efficacy of cellular therapies specifically hematopoietic cell transplantation (HCT) and chimeric antigen receptor T (CART) adoptive cell transfer are summarized in Table 4.

**Table 4:** Validated blood biomarkers for cellular therapies

Biomarker discovered	Biomarker type	Study/Year	*n*	Detection platform	Associations/Timepoints (D0 = HCT or ACT date)	Phase	Ref.
**Hematopoietic Cell Transplantation**							
**GVHD**							
**Panel: IL-2-R-α**,** HGF**,** IL-8**,** TNFR-1**	Diagnostic	Paczesny 2009	424	MS/MS followed by ELISA	Identified acute GVHD at onset of symptoms	Discovery and clinical validation	[49]
**REG3α**	Diagnostic	Ferrara 2011	1014	MS/MS followed by ELISA	Identified GI-GVHD at onset of symptoms	Discovery and clinical validation	[60]
**TIM3**	Prognostic	Hansen 2013; Abu Zaid 2017	149; 211	MS/MS followed by ELISA	Identified GI-GVHD at onset of symptoms, Prognostic for NRM	Discovery and clinical validation	[62, 63]
**ST2 or IL1RL1**	Risk and predictive	Vander Lugt 2013	673	MS/MS followed by ELISA	Increased levels in SR-aGVHD and elevated at D14 post-HCT in patients with high NRM	Analytical and clinical validation	[64]
**Panel: ST2 + REG3α**	Risk and prognostic	Hartwell 2017; Hotta 2021	1287; 112	ELISA	At D + 7 post-HCT categorizes into high and low NRM	Clinical validation	[65, 66]
**Endothelial Activation and Stress Index (EASIX)**	Prognostic	Luft 2017	311, 141,173,89	Clinical practice laboratory tests	Prognostic of NRM and OS	Clinical validation	[67, 68]
**Panel: ST2**,** MMP3**,** CXCL9**,** OPN**	Risk and diagnostic	Yu 2016	391; 172	MS/MS followed by ELISA	Identified chronic GVHD at onset of symptoms, and elevated at D100 post-HCT in patients who will develop cGVHD	Discovery, analytical and clinical validation	[51]
**CD163**	Risk and diagnostic	Inamoto 2017	127	ELISA	Identified cGVHD at onset of symptoms, and D80 post-HCT	Clinical validation	[70]
**Panel: cellular and plasma biomarkers**	Diagnostic	Cuvelier 2023	234 (Pediatric)	Flow cytometry and ELISA	Diagnostic classifier for pediatric cGVHD	Clinical validation in pediatric population	[71]
**DKK3**	Diagnostic	Inamoto 2020	186	MS/MS followed by ELISA	Identified chronic GVHD at onset of symptoms	Discovery	[72]
**Panel: CXCL9**,** MMP3**,** DKK3**	Risk	Logan 2023	982	MS/MS followed by ELISA	Elevated at D100 post-HCT in patients who will develop cGVHD	Clinical validation	[73]
**Leukemia**							
**Minimal Residual Disease (MRD)**	Diagnostic	Wong 2023 (review)	NA	Molecular and flow cytometry	Recommendations: MRD testing prior to HCT can estimate relapse and death post-HCT, Molecular testing better than flow cytometry	Clinical application	[74]
**TP53 mutation (MDS)**	Diagnostic	Lindsley 2017	1514	Sequencing	TP53 mutations were present in 19% of the patients, and were associated with shorter survival, and a shorter time to relapse than was the absence of TP53 mutations	Clinical application	[75]
**GVL**							
**61 proteins- signature**	Prognostic	Liu 2019	17	MS/MS followed by scRNAseq	unique 61-protein signature of GVHD-free GVL	Discovery	[56]
**CART cell Therapies**							
**CRS/ICAN/cytopenias**							
**IL6** **(meta-analysis**,** systematic review)**	Risk	Tedesco 2021, Biery 2024	NA	Immunoassays	Review conclusions: Highest levels worst toxicities, Early timepoints more severe toxicities, Need for standardization of assays used	Clinical application without Analytical validation of a single test	[77, 79]
**Baseline CRP** + **ferritin**	Risk and Prognostic	Faramand 2024	136	Clinical practice laboratory tests	Correlates with baseline IL6, 3 groups risk stratification for prognosis of OS	Clinical validation	[78]
**EASIX-pre (infusion)**	Risk	Korell 2022	214	Clinical practice laboratory tests	Correlates with D7 EASIX, Associated with grade ≥ 3 CRS and/or ICAN	Clinical validation	[79]
**CAR-HEMATOTOX**	Risk	Rejeski 2021	458	Clinical practice laboratory tests	Acquired at baseline, Includes markers associated with hematopoietic reserve (e.g., platelet count, hemoglobin, and ANC), and baseline inflammation (e.g., C-reactive protein and ferritin), Associated with delayed cytopenia	Clinical validation	[81]
**Tumor**							
**Baseline tumor burden and characteristic**	Risk	Cottrell 2025	NA	Pathology	SITC Consensus Table 2 has essential biomarkers for tumor burden	Clinical application	[82]
**Response to lymphodepletion**	Risk	Hirayama 2019	48	ELISA	Higher IL-7 and MCP-1after lymphodepletion are associated with better PFS	Clinical validation	[83]
**MRD post-CART**	Prognostic	Hay 2019; Bansal 2023	53; 60	Molecular and flow cytometry	In lymphoma patients, MRD-negative complete remission 3 weeks after CAR T-cell infusion is associated with improved overall and event-free survival, In myeloma patients, MRD-negative status at month 1 post-CART is correlated with response and prolonged progression-free survival	Clinical validation	[84, 85]
**Antigen loss**	Prognostic	Mishra 2024	NA	Sequencing	Antigen loss leads to relapse or treatment failure in nearly 30–70% of patients	Clinical application	[86]
**Second tumor**	Diagnostic	Hamilton 2024, Dulery 2025	724, 3066	Pathology	Stanford: 1/724 case of T cell malignancy associated with DNMT3A and TET2, and 25/724 second tumors DESCAR-T: 1/3066 case of T cell malignancy by integration	Clinical application	[87, 88]
**CART efficacy**							
**CART expansion and persistence**	Diagnostic	Blumenberg 2023, Turicek 2023	126, NA	Sequencing	Conclusions: Monitoring of CAR T-cell kinetics outside of clinical trials has largely been overlooked Flow-based assay would enable treatment failure to be detected earlier, and accelerate the widespread clinical application	Clinical application	[89, 90]
**InflaMix**	Risk	Raj 2025	688	Clinical practice laboratory tests	InflaMix revealed an inflammatory signature associated with a high risk of CAR-T treatment failure, including increased hazard of death or relapse (hazard ratio, 2.98; 95% confidence interval, 1.60–4.91; *P* < 0.001).	Clinical validation	91
**Memory phenotype and Exhaustion markers**	Predictive	Fraietta 2018, Deng 2020	41, 24	Flow cytometry	Lower frequencies of PD1 or exhaustion signature expressed by infused and engrafted CAR-T cells are correlated with better disease control	Clinical validation	[92, 93]
**CAR T**_**Reg**_ **cells**	Prognostic	Good 2022	32	Flow cytometry	High CAR T_Reg_ cells associate with clinical progression and less severe neurotoxicity	Discovery	[94]
**High IFN signaling and TME MDSCs**	Prognostic	Jain 2021	105	Sequencing and Flow cytometry	Poor CAR T-cell expansion is associated with tumor IFN signaling and peripheral blood M-MDSCs	Discovery	[95]

Footnotes: aGVHD, acute graft versus host disease, cGVHD, chronic graft versus host disease, SR, steroid refractory, NRM, nonrelapse mortality, OS, Overall Survival, EASIX, Endothelial Activation and Stress Index, CRP, C-reactive protein, InflaMix, INFLAmmation MIXture Model

#### Biomarkers in hematopoietic cell transplantation

Allogeneic hematopoietic cell transplantation (HCT), also known as allogeneic stem cell transplant, is widely considered the most validated cellular therapy because it is the most common form of cellular immunotherapy and has been widely available for over 60 years. With over one million transplants performed worldwide, it has paved the way for advancements in stem cell therapies, immune-modulating techniques, and personalized cancer therapeutics such as CART adoptive transfer. Similarly, development of proteomics biomarkers for acute and chronic GVHD, two major complications following HCT have allowed a better understanding of GVHD and pathed the way for biomarkers for other cellular therapies [64, 65]. A biomarker panel of IL-2 Receptor-α (IL-2Rα), tumor necrosis factor receptor-1 (TNFR-1), interleukin-8 (IL-8) and hepatocyte growth factor (HGF) obtained at the onset of the clinical symptoms was able to confirm acute GVHD at diagnosis [55]. Reg3α, a peptide primarily found in Paneth cells of the gastro-intestinal (GI) tract has emerged as the most validated biomarker of acute GVHD of the GI tract [66, 67]. The soluble form of T-cell immunoglobulin mucin protein-3 (TIM3) was also associated with acute GVHD of the GI tract [68]. At 28 days post-HCT, TIM3 concentrations in addition to ST2, correlated with 2-year NRM [69]. Stimulation-2 (ST2), or IL1RL1 (new name based on the HUGO nomenclature) is the interleukin-33 (IL-33) decoy receptor, and it is involved in inflammatory signaling. Following HCT, IL1RL1 is the most validated biomarker for acute GVHD and has been studied in a variety of clinical scenarios. When ST2 was measured at the start of corticosteroid treatment, patients with high ST2 were over twice as likely to have treatment resistant acute GVHD [56]. ST2 and Reg3α measured at 7 days post-HCT were used to create a prognostic algorithm that separate patients into groups with significantly different 6-month NRM [70]. An Endothelial Activation and Stress Index (EASIX) has been developed and is calculated using lactate dehydrogenase (LDH), creatinine, and platelet count. It has been associated with increased risk of acute GVHD and mortality in retrospective and prospective cohorts [71, 72]. A 4-biomarker panel consisting of ST2, CXCL9, matrix metalloproteinase-3 (MMP-3), and osteopontin (OPN) had significant correlation with chronic GVHD at diagnosis and 100 days post-HCT [57]. CD163 is a scavenger receptor shed by activated monocytes/macrophages during times of oxidative stress [73]. CD163 concentrations at 80 days post-HCT were associated with *de novo* chronic GVHD [74]. In a pediatric cohort of 234 recipients, a classifier including decreases in regulatory natural killer cells, naïve CD4 T helper cells, and naïve regulatory T cells, and elevated levels of CXCL9, CXCL10, CXCL11, ST2, ICAM-1, and soluble CD13 (sCD13) characterized the onset of chronic GVHD [75]. Dickkopf-related protein 3 (DKK3), a modulator of Wnt signaling involved in fibrosis, has been identified as a potential chronic GVHD diagnostic biomarker of sclerotic skin chronic GVHD by MS/MS proteomics, but was also elevated with other forms of chronic GVHD [76]. In a recent study for risk biomarkers of chronic GVHD, it was elevated in a panel including CXCL9, MMP3 and DDK3 at D100 post-HCT in patients who will develop future occurrence of chronic GVHD in cohorts totaling 982 patients [77].

Relapse of the primary disease remains the main cause of death post-HCT and monitoring minimal residual disease (MRD) is critical as it has been reviewed recently by Wong and colleagues [78], particularly if the patient carry TP53 mutations which are present in 19% of the patients, and are associated with shorter survival, and a shorter time to relapse as compared to HCT recipients without TP53 mutations [79]. One study used an intact-protein analysis system to profile the plasma proteome post- donor lymphocyte injection (DLI) of patients who experienced GVL and acute GVHD for comparison with the proteome of patients who experienced GVL without GVHD [62].

#### Biomarkers in CART cells therapies

Novel genetically engineered immunotherapies have improved the outcomes for patients with advanced hematologic malignancies. As of 2025, seven FDA-approved CAR-T therapies are available. Utilizing and standardizing biomarkers of toxicity and efficacy of these novel CART cells therapy will allow for better management of side effects and prediction of treatment outcomes. Identifying biomarkers can help predict patient-specific responses to treatment, including the risk of severe side effects like cytokine release syndrome (CRS) and neurotoxicity, as well as the likelihood of achieving a complete remission [80–82]. Here, we will focus on the most validated biomarkers in the field so far and that are also summarize in Table 4.

The 3 major toxicities of CART cells therapies are cytokine release syndrome (CRS), Immune Effector Cell-Associated Neurotoxicity Syndrome (ICAN), and cytopenia. Plasma IL6 has been the most validated cytokine related to CRS/ICAN with high levels at earlier timepoints post-infusion correlated with the worst toxicities. The IL6 clinical assay must be standardized across studies and clinical laboratories [81, 83]. A baseline combined C-reactive protein (CRP) and ferritin formula correlated with baseline IL6 and risk stratification in 3 groups (low, intermediate, high) was prognostic of overall survival [82]. The EASIX formula pre-infusion correlated with the day 7 post-infusion and associated with grade ≥ 3 CRS and/or ICAN [84]. For cytopenias, Rejeski et al. developed the CAR-HEMATOTOX model at baseline which Includes markers associated with hematopoietic reserve (e.g. platelet count, hemoglobin, and ANC), and inflammation (e.g. CRP and ferritin) correlated with delayed cytopenia [85]. For biomarkers relevant to the tumor evaluation, like for hematopoietic cell transplantation, the baseline tumor burden and characteristic are important parameters and essential biomarkers have been summarized in the recent Society for Immunotherapy of Cancer (SITC) consensus by Cottrell et al. [86]. Biomarkers of the response to the lymphodepletion such as higher IL-7 and MCP-1 have been associated with better progression-free survival (PFS) [87]. A negative MRD 3 weeks following CAR T cells infusion is associated with improved overall survival (OS) and event-free survival (EFS) or PFS in lymphoma and myeloma patients [88, 89]. Antigen loss on the tumor is also an important biomarker of relapse or treatment failure [90]. Finally, in extremely rare cases (< 0.1%) a T cell malignancy due to insertion has been observed while non-insertional second tumors are seen in 3–5% [91, 92]. Several biomarkers of CART cell efficacy have been developed. The most established way to monitor CART efficacy is to follow CART expansion and persistence with CART peak in % of T cells or in cells per microliter detected by either flow cytometry or molecular assays being the most used. However, there is a large variability if CART-cell detection and establishing a standardized approach for collecting and reporting data is urgently needed [93, 94]. A recent study used an INFLAmmation MIXture Model (InflaMix) including 14 pre-CART infusion laboratory and cytokine measures capturing inflammation and end-organ function, and correlated with outcomes including CART treatment failure, relapse, death. High InflaMix was associated with increased hazard of relapse and death probable reflecting the status of the patient and tumor rather than the efficacy of the CART therapy itself [95]. Higher frequencies of markers of memory phenotype and lower frequencies of T cell exhaustion on CART cells in the product are correlated with better disease control [96, 97]. In contrast, high CAR T_Reg_ cells in the product associated with clinical progression but less severe neurotoxicity [98]. Parameters from the tumor microenvironment are also emerging as critical, for example high IFN signaling and myeloid-derived suppressor cells (MDSCs) in the tumor microenvironment is associated with poor CART cells expansion [99].

### Evaluation of biomarker performance and novel approaches to improve it

#### Receiver operating characteristic (ROC) curves

**Fig. 3:**
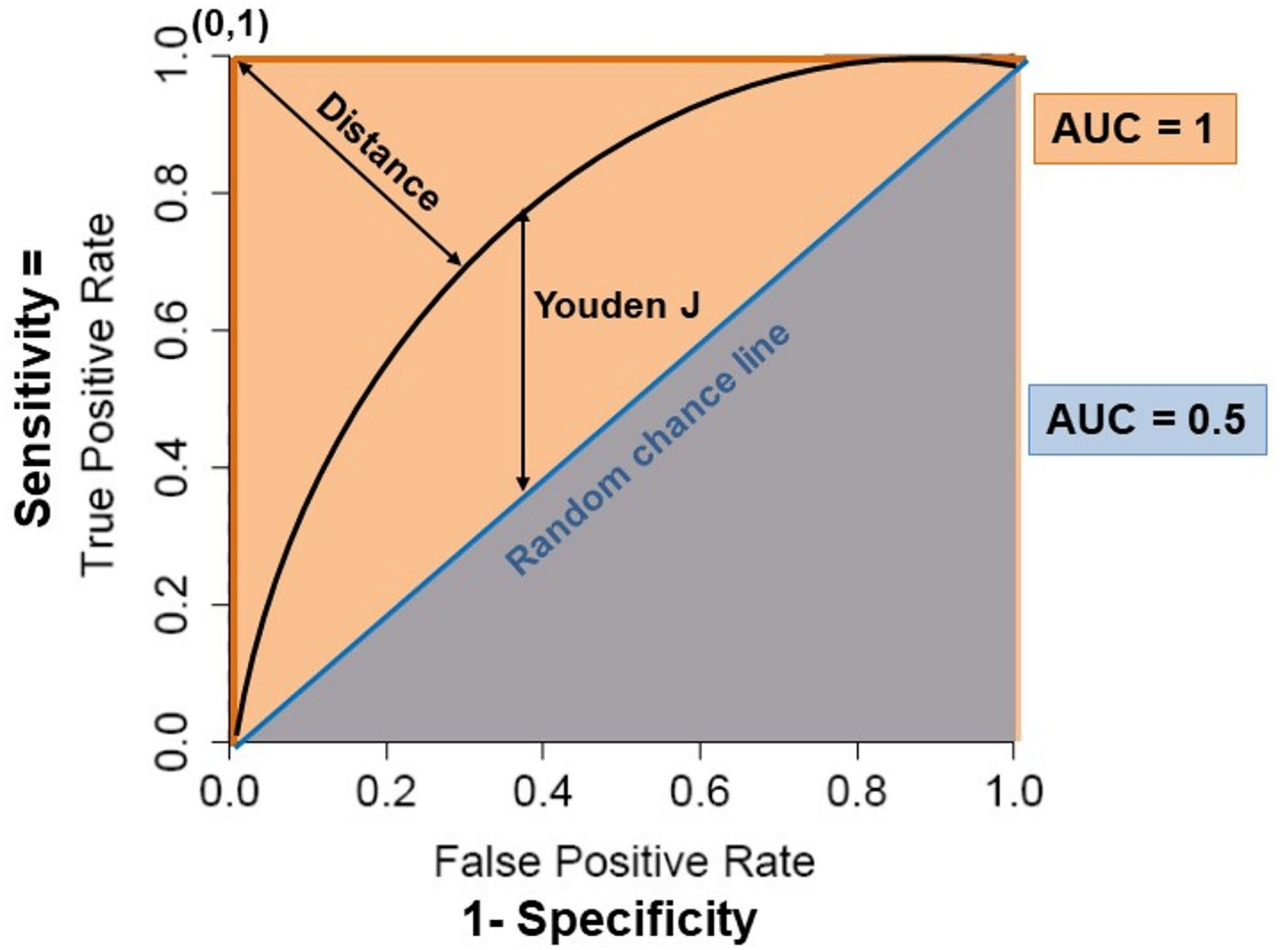
Area Under the Receiver Operating Characteristic (AUROC) Curve. AUROC curve is a metric used to evaluate the performance of a biomarker. It represents the False Positive Rate (1-Specificity) on the x-axis versus the True Positive Rate (Sensitivity) for every threshold of the biomarker). An AUC of 0.5 is on the random chance line and an AUC of 1.0 is a perfect test with 100% specificity and 100% sensitivity. The Youden’s index selects as a threshold for the biomarker value that maximizes lift of the ROC curve from the diagonal. Another way to find the optimal threshold is to identify the point on the ROC curve closest to the (0,1) corner (the “perfect” classifier point) which minimizes the Euclidean distance between the curve and (0,1). This point represents a balance between sensitivity and specificity

Sensitivity is the percentage of subject with a disease who are correctly identified by a test; specificity is the percentage of subject without a disease who are correctly excluded by a test. The area under the curve (AUC) of ROC (AUROC) is the most objective metric to evaluate the performance of binary classifiers [100]. A ROC curve is a graphical tool that assesses a biomarker’s performance by evaluating its 1-sensitivity on the x-axis and specificity on the y-axis for every possible cutpoints of a biomarker. The AUROC summarizes the overall accuracy of the test with a higher AUC value, a higher accuracy. A right angle will be a perfect test with AUC of 1 meaning 100% sensitivity and 100% specificity while the random chance line will indicate an AUC at 0.5. Importantly, ROC curves are non-parametric, thus they don’t require the predicted positive or negative score distributions to be normally distributed. This makes ROC curves less dependent on the prevalence of a given outcome than other performance metrics. From the ROC curve, identification of optimal cutpoints uses two common methods: the Youden’s J statistic also called Youden’s index where J is the maximum vertical distance from the curve to the chance line or diagonal [101], and the closest to the (0,1) point on the ROC [102, 103]. Although both maximizes sensitivity and specificity, they might pick a different cutpoint. A simplified schema of ROC curve and cutpoint evaluations is shown in Fig. 3.

#### Improvement of sensitivity and specificity of biomarkers with machine learning (ML) approaches

**Fig. 4:**
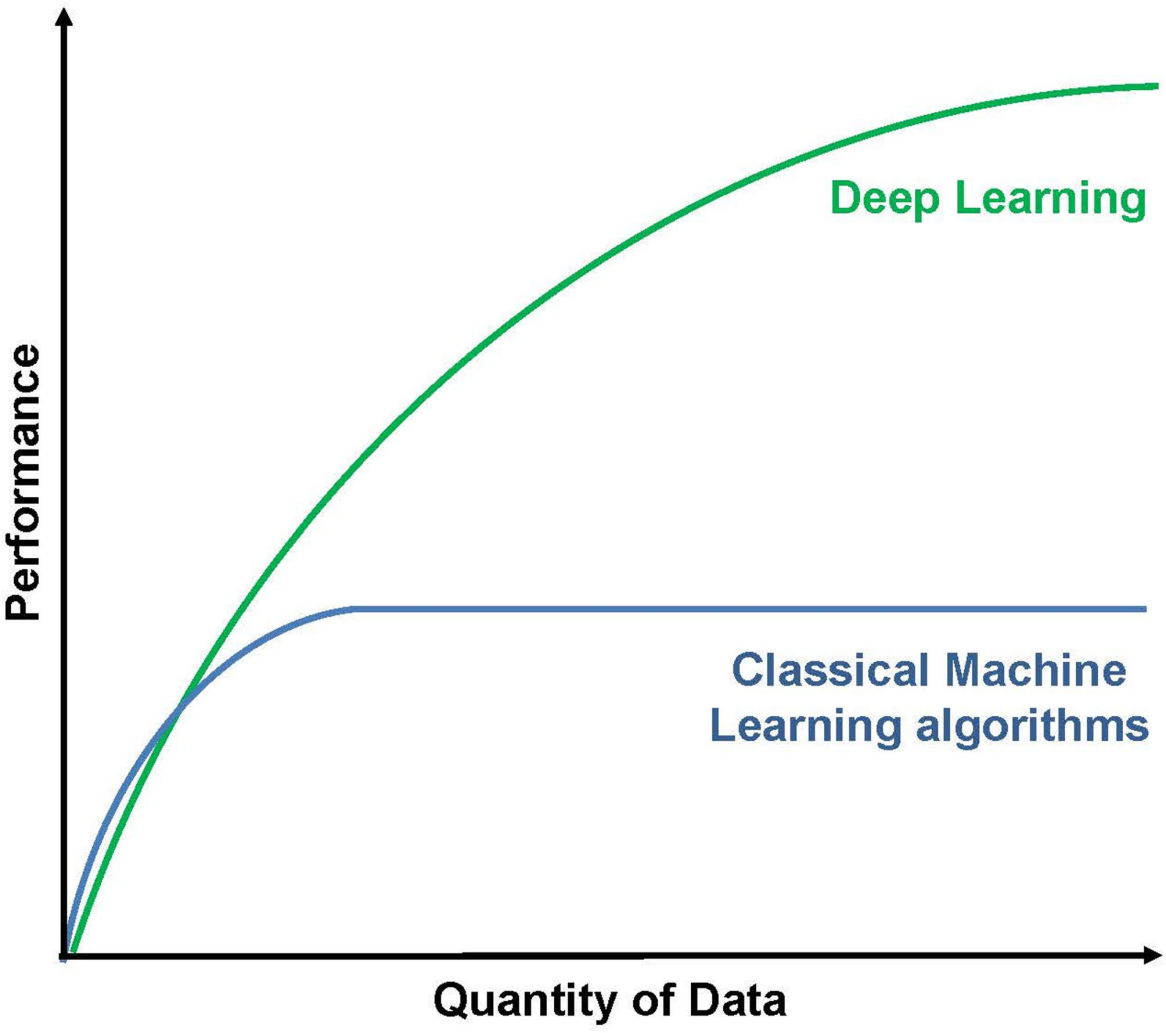
Deep Learning Performance Improves with More Data Compared to Traditional Algorithms. This figure illustrates how the performance of different data science algorithms scales with increasing amounts of training data. Deep learning algorithms show a strong positive correlation between data volume and performance, outperforming traditional machine learning methods as dataset size grows. This scalability is a defining advantage of deep learning in data-rich environments

Machine Learning (ML) techniques adapt to the data to better capture complex nonlinear relationships and interactions between high-dimensional covariates that are difficult to handle with traditional regression. Three broad supervised ML strategies should be considered. Approach (1), which is within the Cox proportional hazards (PH) model framework including Boosting (XGBoost), Group SCAD, and Adaptive Group Lasso [104]. These are data-driven diverse logistic regression ensembles and are closer to classical clinical statistical models. Approach (2), which is termed Tree Ensembles and includes Random forests, Boosting, and Bayesian Additive Regression Trees (BART), aggregates multiple trees for prediction to capture real relationships in the data while the noise in the tree fit washes out on the average but lose the interpretability of single tree [105–108]. These methods avoid the PH assumption. Approach (3) termed Deep learning [Large trained Neural network] uses automatic feature extraction at multiple levels of extraction and allows a system to learn complex functions mapping the input (X) to the output (Y) directly from data, without relying on a “model” [109]. This approach is compared to artificial intelligence because it provides machines the capability to automatically learn and improve from experience without being explicitly programmed. It is primarily focusing on predictions, automatically identifying patterns within data, and performing tasks beyond human capabilities. It is important to note that deep learning results get better with more data and more computation (Fig. 4), which may be a limitation in cohorts from rare diseases such as GVHD or CRS or ICAN following cellular therapies. ML techniques are widely used nowadays in the healthcare domain for the diagnosis, prognosis, and evaluation of treatments, and have applications in the field of HCT. A recent systematic review of the application of ML techniques in the HCT setting was conducted; 27 studies met the inclusion criteria [110]. Of those 63% used ensemble learning, 44% regression, 30% Bayesian learning, and 30% support vector machine. Most studies examined models to predict HCT outcomes (e.g., survival, relapse, GVHD…). Clinical and genetic data were the most used predictors in the modeling process. The authors concluded that, so far, the evidence is not sufficiently robust to determine the optimal ML technique to use in the HCT setting and/or what minimal data variables are required. Thus, this is still a gap of knowledge particularly for ML-defined biomarkers in the cellular therapy field. In addition, none of these studies, so far, have included plasma biomarkers collected following treatment with cellular therapy. One promising pilot study revealed novel chronic GVHD clinical phenotypes at diagnosis that also stratified survival [111].

### Clinical implementation

**Fig. 5:**
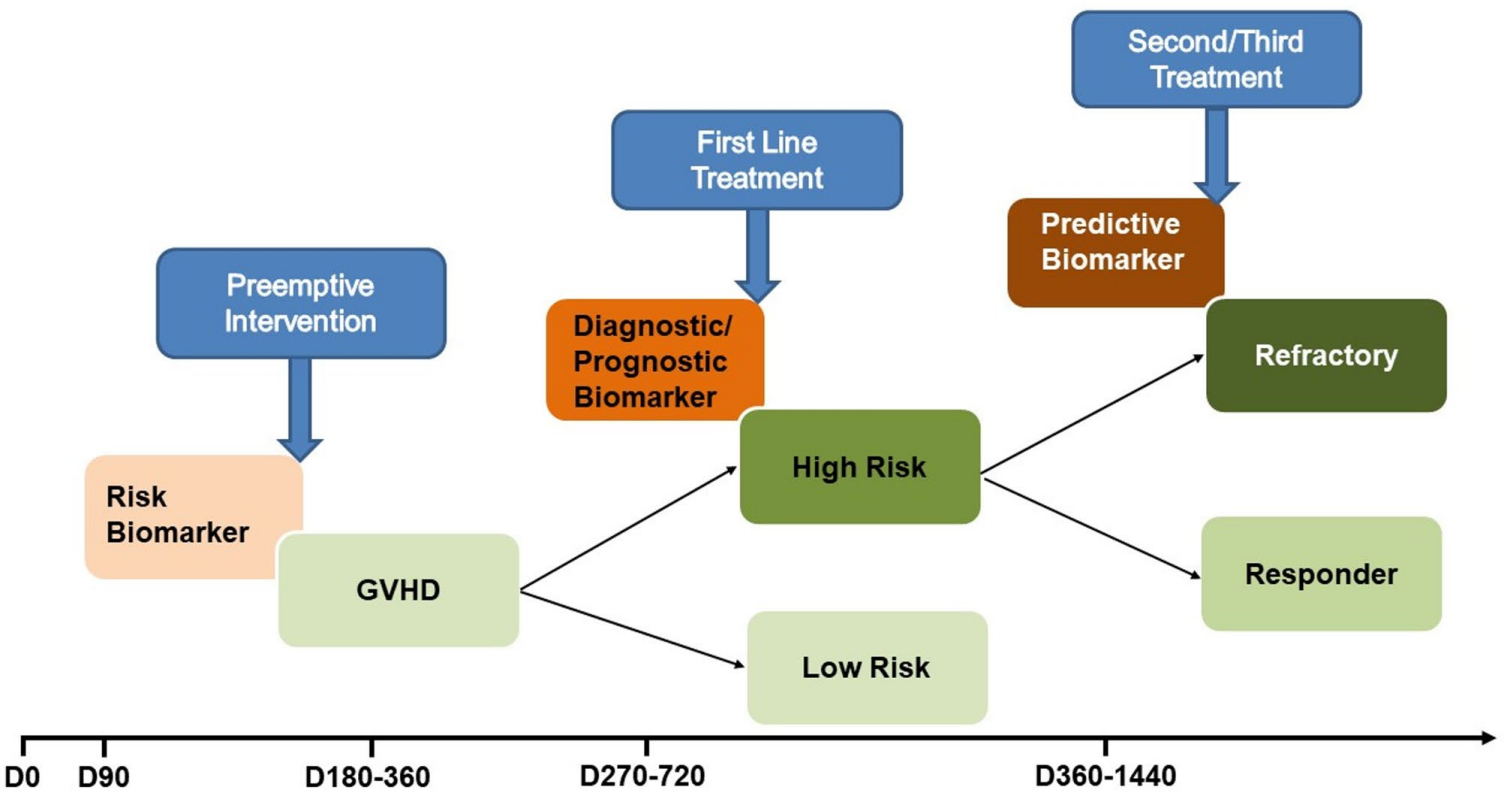
Implementation To the Clinic. Proposed clinical interventions guided by biomarker type and chronology following allogeneic hematopoietic cell transplantation (HCT). This approach aims to move towards personalized medicine, where treatment decisions are tailored based on individual patient characteristics and biomarker profiles. Risk biomarker of acute (day 14 post-HCT) or chronic GVHD (day 90 or 100 post-HCT) enable preemptive trials. Diagnostic and prognostic biomarkers play a significant role in influencing treatment intensification in high values or rapid immunosuppression taper with low values. Predictive biomarkers allow to identify which patients are most likely to respond to specific therapies and therefore are essential for personalized treatment

Actionable biomarkers are biomarkers for which results of the testing can be used to guide clinical management of disease. As mentioned earlier, it is important to utilize the correct type of biomarker for the outcome in question. In the field of cellular therapies, the intervention is dependent on the time following HCT. Diagnostic biomarkers could for example distinguish patients with GVHD from those without GVHD or with a specific target organ GVHD (i.e. BOS vs. infectious pulmonary). A predictive biomarker for GVHD therapy allows to intensify the treatment in the high-risk patients and decrease immunosuppression in low-risk patients. Response biomarkers enable monitoring of the pharmacodynamic of a specific treatment. In view of the newer prophylaxis that significantly reduce GVHD (i.e. post-cyclophosphamide post-transplant, Orca-T) but currently given to all comers, it will be beneficial (for engraftment, infectious, and GVL outcomes) to target only the higher risk patients. This goal of risk-adapted strategy can be achieved by using a risk biomarker-based preemptive trial focusing on the high risk-patients. Figure 5 summarizes how to harness clinically the different type of biomarkers in the HCT setting.

### Conclusions and future directions

Proteomic data at scale have only recently become accessible to large-scale proteogenomic approaches, largely due to previous analytical limitations with MS-approaches specific to the composition of the plasma proteome. Advances in affinity proteomics technologies are rapidly correcting this shortfall, with recent studies reporting the parallel measurement of > 5,000 proteins in > 10,000 blood samples [4], and other large studies under way [112]. Recent advances in the scale and scope with which it is possible to survey the plasma and other biofluids proteome open new opportunities to use proteomics to deliver improved understanding of the mechanistic basis of disease [113] and to promote novel translational strategies through target and biomarker identification. In the field of cellular therapies, discovery of novel candidate biomarkers employing proteomics has provided several promising candidates and some have already been implemented, but biomarker validation and standardization across institutions in prospective and interventional trials need to be expanded. Biological samples from patients receiving cellular therapies should be collected and reported in a standardized manner. Network development for collaboration between clinicians, academia, pharmaceuticals, and health authorities is essential with focus on centralized biorepositories with standardized sample storage and data analysis. By expanding the number of validated biomarkers, researchers could design prospective, randomized studies leading to an increase in risk-adapted clinical trials. The American Society of Hematology Taskforce for Immunotherapies workshop on biomarker development published an outlined consensus detailing the efforts outlined above [80], with the goal for extension to international registries and plan for future meetings.

## Data Availability

Not applicable.
